# Toward equity in smoking cessation: evidence from Saudi Arabia’s 2022 Global Youth Tobacco Survey through the Lens of Levesque’s Access-to-Health-Care Framework

**DOI:** 10.3389/fpubh.2026.1914890

**Published:** 2026-09-23

**Authors:** Sulafa T. Alqutub

**Affiliations:** Department of Family and Community Medicine, College of Medicine, University of Jeddah, Jeddah, Saudi Arabia

**Keywords:** adolescent health, GYTS 2022, health equity, health services accessibility, Saudi Arabia, smoking cessation

## Abstract

**Purpose:**

The 2022 Saudi Global Youth Tobacco Survey (GYTS) provided information on smoking status, quitting attempts, and help received for cessation among 6,983 eligible intermediate school students. Cessation services are available for free; however, still they are underutilized by smokers. Using data from the 2022 Saudi GYTS, this study examined the determinants of inequities in access to smoking cessation services and assessed the gap between policy intent and real-world access within the Saudi system.

**Patients and methods:**

A complex sample design analysis was used to assess access to smoking cessation services among youth while accounting for survey weighting, clustering, and stratification, using Levesque et al.’s framework, focusing on the demand side. Regression analysis was applied to control for confounders.

**Results:**

Subgroup analyses were conducted on 495 current tobacco users aged 13–15 years from the 2022 GYTS data, representing an estimated weighted population of approximately weighted *N* = 101,378 youth. Of these, 23.1% reported receiving help to quit or advice indicating that they should quit, and 79.3% reported making a quitting attempt over the previous 12 months. Uncertainty or lower perceived harm regarding exposure to others’ smoking was independently associated with greater odds of receiving help or advice to quit. Younger adolescents, particularly 13-year-olds, had higher odds of reporting a quit attempt than older tobacco users.

**Conclusion:**

Even though 23.1% reported receiving some form of help quitting, still 20.7% did not report quitting attempt. This pattern raises concerns with respect to passive service models that rely on individual willingness. Effective tobacco control efforts should go beyond passive service availability, integrating proactive, school-based cessation support, clear harm-awareness messaging, and outreach targeting especially uncertain older tobacco users’ youth. Ensuring equitable access requires early, structured, and tailored interventions that can actively engage adolescents before they seek help. Findings underscore the importance of improving youth-centered cessation surveillance, access measurement, and service equity to better understand social and service-related barriers.

## 1 Introduction

Studies of the global burden of disease show that smoking causes nearly 8 million deaths annually and reduces disability-adjusted life years by 200 million worldwide, and is consistently one of the leading drivers of global morbidity and premature mortality (1). Tobacco smoking is a modifiable behavioral risk that requires effective policy-level and individual-level interventions (2). In Qatar, young smokers with higher incomes and higher educational attainments are part of the higher-risk group, requiring tailored interventions to help them quit smoking (3). Globally, smoking onset is common among youth aged 13–15 years. In 2013, a health information survey conducted in Saudi Arabia revealed that approximately 30% of Saudi citizens began smoking before the age of 15 years, and 60% started smoking before the age of 18 years (4).

The most recent data from the Saudi Global Youth Tobacco Survey (GYTS) (2022) contain information on smoking status, quitting attempts, and help received for smoking cessation from 6,983 intermediate school students. The prevalence of tobacco product use declined from 26.1% in 2007 to 9.4% in 2022, representing a substantial reduction over the survey period. In 2007, the overall rate of quitting attempts among teenagers aged 13–15 years over the previous 12 months was 62.3%, and the rate of receiving help to quit smoking was 78.5% (5, 6). Relative to the data from the 2007 survey, quit attempts were slightly greater over the following decades, reaching 64.9% by 2022. Furthermore, the proportion of youths who received help to quit smoking showed a marked decline, with a 57.1-percentage-point absolute reduction, reaching 21.4% (6, 7).

The Saudi Ministry of Health (MOH) reported that 4,697 online visits for smoking cessation had taken place by 2021. Likewise, in 2022, 630 smoking cessation clinics were established across 20 health regions, of which 100 were mobile cessation clinics that were distributed across the country (6, 8, 9). In the Saudi health system, smoking cessation services are provided for free, with 60% of smokers being aware of these services; however, only around 9% of smokers utilize them (10). One reason for this is the rise in the popularity of alternative tobacco products, such as e-cigarettes (11)—approximately 5.4% of the youth surveyed reported vape consumption (12). Multiple studies have been conducted worldwide to evaluate whether smoking cessation protocols that have been developed for adults can effectively lead to abstinence among youths who smoke using either tobacco or nicotine vaping products (13, 14). Monshi et al. (11) identified a clear research gap and a lack of comparable data on youth-specific global smoking cessation experiences, especially regarding quit attempts, success rates, and access to services.

The Access-to-Health-Care Framework developed by Levesque et al. initially defines access to care as the opportunity to identify, seek, reach, obtain, or use health care and to ensure that the need for the service is met; originally, this framework was developed to strengthen the interactions between service design from the supplier side and the population’s capacity from the demand side (15, 16).

The Saudi GYTS provides indirect information on access to cessation services in relation to demand (6). The Saudi GYTS provides indirect evidence of demand for cessation services through indicators such as intention to quit and previous quit attempts, which reflect a potential need for cessation support; however, it does not directly assess access to or utilization of formal health services for tobacco cessation. The survey provided smoking-related data on youth regardless of their smoking status. However, the available country-specific tobacco control profile does not provide evidence concerning the pattern of quitting attempts, abstinence periods, and nicotine vaping product use as a method of cessation among youth (17). This information is required to determine and plan individualized management within this specific age group. However, across a range of countries, such as Canada, the UK, Australia, and the US, most quit attempts are made without assistance and likely without utilizing government-approved cessation medications (18).

This study investigated potential sociodemographic inequities in relation to smoking cessation services among tobacco users participating in the Saudi GYTS and assessed the gap between policy intent and real-world access for youths in the context of the Saudi system. This analysis provides support for the identification of risk exposure pathways and factors related to both help received to quit smoking and quitting attempts.

## 2 Materials and methods

### 2.1 Study design and data source

This is a secondary analysis of the 2022 Saudi Arabia Global Youth Tobacco Survey (GYTS), a cross-sectional, nationally representative, school-based survey of intermediate-school students conducted jointly by the Ministry of Health and the Ministry of Education. GYTS employs a standardised two-stage cluster sample design in which schools are selected with probability proportional to enrolment size and intact classes are then chosen at random, with all students in the selected classes eligible to participate. Data are collected with an anonymous, self-administered, scannable paper questionnaire. In 2022, 6,983 eligible students in the first to third intermediate grades completed the survey (overall response rate 92.3%); in keeping with GYTS convention, estimates are reported for students aged 13–15 years (*n* = 5,610).

### 2.2 Study population

The present analysis was restricted to current tobacco users aged 13–15 years—students who reported using any tobacco product (manufactured cigarettes, shisha or other smoked tobacco, electronic cigarettes, heated tobacco, and/or smokeless tobacco) on one or more of the 30 days preceding the survey. This produced an analytic sample of 495 current tobacco users, representing a weighted population of approximately 101,378 students, distributed across 46 sampling strata (SS) and 48 primary sampling units (PSU).

### 2.3 Conceptual framework

Demand-side determinants of access to cessation support were organized using the Levesque et al. (15) patient-centered model of access, which specifies five population abilities that mirror the supply-side dimensions of accessibility: the ability to perceive, to seek, to reach, to pay, and to engage. Each GYTS indicator used in this study was mapped *a priori* to one of these abilities (Table 1). Tobacco accessibility and affordability were used as contextual proxy indicators of the broader structural environment surrounding tobacco use, rather than as direct measures of accessibility to cessation services.

**Table 1:** Sociodemographic characteristics of current tobacco users aged 13–15, 2022 Saudi GYTS (*N* = 495).

Characteristic	Category	*n* (unweighted)	Weighted % (95% CI)
Gender	Male	294	58.1 (48.1–67.4)
	Female	201	41.9 (32.6–51.9)
Age (years)	13	145	29.8 (24.0–36.4)
	14	182	35.8 (30.5–41.5)
	15	168	34.4 (28.9–40.4)
Intermediate grade	First	133	27.6 (22.3–33.6)
	Second	184	34.7 (29.3–40.4)
	Third	178	37.7 (32.2–43.5)

Percentages are survey-weighted; *n* values are unweighted counts.

### 2.4 Outcome variables

Two cessation outcomes were examined, each defined to capture quit behavior across all tobacco products used by the respondent rather than cigarettes alone: (i) any attempt to stop using tobacco during the past 12 months (yes/no) and (ii) ever having received help or advice to stop from a Programme or health professional (yes/no). Each outcome was derived by pooling the product-specific cessation items, so that a participant was classified as “yes” if the behavior was reported for any product they used. Under this definition the two outcomes were available for 186 and 189 respondents, respectively. It is worthening that, because the selected outcomes pool across all consumed product types, their estimates (79.3% ever tried to stop; 23.1% received help) are higher than the WHO/CDC fact-sheet figures, which are restricted to current smokers (64.9 and 21.4%) (7). So, this should not be read as a discrepancy.

### 2.5 Explanatory variables

Demand-side ability indicators comprised: exposure to anti-tobacco messages in the media or at community events, having been taught about the dangers of tobacco in class, and definitely perceiving other people’s smoking as harmful to reflect on (ability to perceive); and the average amount of money spent per week, dichotomized as less than 30 SAR versus 30 SAR or more to reflect on (ability to pay). Sociodemographic covariates were age (13, 14 or 15 years), gender, and intermediate grade (first, second or third).

### 2.6 Statistical analysis

Data were analyzed using IBM SPSS advanced statistics (Statistical Package for Social Sciences), version 27 (Armonk, NY: IBM Corp). All analyses accounted for the complex survey design using the final sampling weight, the sampling strata and the primary sampling units, with standard errors estimated by Taylor-series linearization, so that estimates are representative of only Saudi students aged 13–15 years old. Categorical variables are summarized as survey-weighted percentages with 95% confidence intervals, alongside unweighted frequencies. The association of each explanatory variable with each cessation outcome was assessed first in univariable survey-weighted logistic regression (crude odds ratios) and then in a multivariable model mutually adjusting for age, gender, grade, pack price, anti-tobacco message exposure and perceived harm (adjusted odds ratios with 95% confidence intervals). Reference categories were 15 years, male, third intermediate grade, average spending below 30 SAR, message-exposed and perceiving harm. Age and grade, although correlated, were both retained so that adjusted estimates could be reported for each; the estimates were stable with no evidence of variance inflation. Analyses were complete-case, and a two-sided *p*-value below 0.05 was considered statistically significant.

### 2.7 Ethical consideration

This study was conducted drawing on secondary analysis of publicly available data from the 2022 GYTS, a nationally representative, school-based survey that was conducted using standardized international protocols by both the MOH and Ministry of Education, receiving ethical approval and implementing informed consent procedures during primary data collection. The dataset used in this study was fully anonymized, so it posed no risk to individual participants. The analysis relied exclusively on de-identified and publicly accessible data; hence, no additional ethical approval or informed consent was required for the secondary analysis. All of the methods adopted conformed to the relevant ethical guidelines and regulations applicable to research involving human participants. An exemption was requested from the local IRB. The approval decision was received under the following project title: Equity in Smoking Cessation: Evidence from Saudi Arabia’s 2022 Global Youth Tobacco Survey through the Lens of Levesque’s Access-to-Health Care Framework with the approval number UJ-REC-422.

## 3 Results

### 3.1 Participant characteristics

The analysis included 495 current tobacco users aged 13–15 years (weighted *N* = 101,378) sampled across 46 strata and 48 primary sampling units. Boys accounted for 58.1% of participants and girls for 41.9%. Participants were distributed across ages 13 (29.8%), 14 (35.8%) and 15 years (34.4%), and across the first (27.6%), second (34.7%) and third (37.7%) intermediate grades (Table 1).

### 3.2 Demand-side access indicators (Levesque framework)

Table 2 presents the demand-side access indicators with survey weights. In the ability-to-perceive domain, 61.3% of participants had been exposed to anti-tobacco messages through media or community events, 43.4% had been taught about the dangers of tobacco in class, and only 35.6% definitely regarded other people’s smoking as harmful. In the ability-to-seek domain, 79.3% of current tobacco users reported at least one attempt to stop using tobacco in the previous 12 months. For ability to reach, among those reporting how they obtained tobacco, 60.3% had accessed it through public commercial outlets (a store, shop, street vendor or the internet) and 39.7% through social or other sources. For ability to pay, 52.4% typically spent less than 30 SAR per week and 47.6% spent 30 SAR or more. In the ability-to-engage domain, 23.1% had received help or advice to quit. Because the two cessation indicators (i.e. any attempt to stop using tobacco during the past 12 months and ever having received help or advice to stop) pool quit behavior across all tobacco product types, they exceed the WHO/CDC fact-sheet figures, which are restricted to current smokers (64.9 and 21.4%) (7).

**Table 2:** Demand-side access indicators among current tobacco users aged 13–15, 2022 Saudi GYTS.

Levesque domain	Indicator	Category	*n*	Wtd %	95% CI
Ability to perceive	Exposed to anti-tobacco messages (media/community)	Yes	305	61.3	54.9–67.3
		No	190	38.7	32.7–45.1
	Taught about the dangers of tobacco in class	Yes	207	43.4	38.1–48.9
		No	266	56.6	51.1–61.9
	Definitely thought others’ smoking is harmful	Yes	171	35.6	29.7–41.9
		No	312	64.4	58.1–70.3
Ability to seek	Attempt to stop using tobacco, any product (12 months)	Yes	148	79.3	72.1–85.1
		No	38	20.7	14.9–27.9
Ability to reach	Source of tobacco, any product	Public/commercial	136	60.3	51.7–68.3
		Social/other	91	39.7	31.7–48.3
Ability to pay	Average amount spent/week	<30 SAR	256	52.4	47.7–57.2
		≥30 SAR	234	47.6	42.8–52.3
Ability to engage	Received help or advice to quit, any product	Yes	44	23.1	16.9–30.7
		No	145	76.9	69.3–83.1

Percentages are survey-weighted; *n* unweighted. Denominators are respondents to each item (anti-tobacco *n* = 495; taught *n* = 473; harm *n* = 483; source/reach *n* = 227; spending *n* = 490; attempt to stop *n* = 186; help *n* = 189). The cessation and source indicators pool responses across all tobacco product types. Also, Several of the indicators presented describe smoking behavior and exposure rather than the health-service access as conceptualized in the Levesque et al. framework.

### 3.3 Factors associated with receiving help or advice to quit

In univariate analysis, the odds of receiving help or advice to quit were higher among 13-year-olds than 15-year-olds (crude OR 2.15, 95% CI 1.03–4.50, *p* = 0.042), among first-intermediate students (crude OR 2.25, 95% CI 1.00–5.05, *p* = 0.049), and—unexpectedly—among those who did not regard others’ smoking as harmful (crude OR 2.46, 95% CI 1.02–5.94, *p* = 0.045). Young people who obtained tobacco from social or other sources, rather than public commercial outlets, had lower odds of receiving help (crude OR 0.33, 95% CI 0.12–0.85, *p* = 0.023); average spending was not associated with receiving help to quit. In the primary multivariable model, not perceiving others’ smoking as harmful remained independently associated with higher odds of receiving help (AOR 3.10, 95% CI 1.19–8.13, *p* = 0.021); anti-tobacco message exposure was borderline (AOR 0.45, 95% CI 0.20–1.03, *p* = 0.060). When source of tobacco was added in a sensitivity model (*n* = 139), its association attenuated (AOR 0.36, 95% CI 0.09–1.42, *p* = 0.144) and no covariate remained significant (Table 3).

**Table 3:** Univariate and multivariable survey-weighted logistic regression for receiving help or advice to quit (Yes = 1).

Predictor	Category	Crude OR (95% CI)	*p*	Adjusted OR (95% CI)	*p*
Age (years)	13	**2.15 (1.03–4.50)**	**0.042**	1.75 (0.50–6.07)	0.379
	14	1.53 (0.57–4.09)	0.393	0.81 (0.25–2.65)	0.731
	15	Ref.	—	Ref.	—
Gender	Female	0.88 (0.40–1.97)	0.760	0.61 (0.15–2.48)	0.491
	Male	Ref.	—	Ref.	—
Intermediate grade	First	**2.25 (1.00–5.05)**	**0.049**	1.72 (0.39–7.67)	0.475
	Second	2.18 (0.89–5.37)	0.089	1.64 (0.52–5.17)	0.397
	Third	Ref.	—	Ref.	—
Average spending	≥ 30 SAR	0.91 (0.46–1.80)	0.787	0.77 (0.38–1.56)	0.464
	< 30 SAR	Ref.	—	Ref.	—
Source of tobacco	Social/other	**0.33 (0.12–0.85)**	**0.023**	0.36 (0.09–1.42)	0.144
	Public/commercial	Ref.	—	Ref.	—
Anti-tobacco messages	No	0.48 (0.22–1.05)	0.067	0.45 (0.20–1.03)	0.060
	Yes	Ref.	—	Ref.	—
Perceived others’ smoking harmful	No	**2.46 (1.02–5.94)**	**0.045**	**3.10 (1.19–8.13)**	**0.021**
	Yes	Ref.	—	Ref.	—

OR, odds ratio; AOR, adjusted odds ratio. Primary multivariable model: *n* = 176 (age, gender, grade, spending, message exposure, perceived harm). The adjusted OR for source of tobacco is from a sensitivity model additionally including it (*n* = 139), in which no covariate was significant; source is reported separately because it was recorded for only 227 participants. Bold: *p* < 0.05. Survey-weighted logistic regression with PSU-clustered (Taylor-linearised) variance. Outcome pools help/advice across all tobacco product types.

### 3.4 Factors associated with a quit attempt in the past 12 months

In the multivariable model, 13-year-olds had markedly higher odds of having attempted to stop using tobacco than 15-year-olds (AOR 4.12, 95% CI 1.04–16.35, *p* = 0.044), an association that emerged only after adjustment and is estimated imprecisely. Higher average tobacco spending (≥30 SAR) was associated with greater odds of a quit attempt but did not reach significance (AOR 1.70, 95% CI 0.86–3.37, *p* = 0.127). Source of tobacco was not associated with quit attempts (crude OR 0.84, 95% CI 0.36–2.00, *p* = 0.701), nor were gender, grade, anti-tobacco exposure or perceived harm (Table 4).

**Table 4:** Univariate and multivariable survey-weighted logistic regression for a quit attempt in the past 12 months (Yes = 1).

Predictor	Category	Crude OR (95% CI)	*p*	Adjusted OR (95% CI)	*p*
Age (years)	13	2.21 (0.63–7.83)	0.218	**4.12 (1.04–16.35)**	**0.044**
	14	1.45 (0.69–3.02)	0.325	1.77 (0.80–3.90)	0.157
	15	Ref.	—	Ref.	—
Gender	Female	1.25 (0.55–2.84)	0.587	0.75 (0.29–1.96)	0.564
	Male	Ref.	—	Ref.	—
Intermediate grade	First	0.83 (0.27–2.60)	0.755	0.46 (0.11–1.95)	0.290
	Second	1.65 (0.67–4.04)	0.274	1.14 (0.39–3.31)	0.806
	Third	Ref.	—	Ref.	—
Average tobacco spending	≥30 SAR	1.40 (0.68–2.90)	0.362	1.70 (0.86–3.37)	0.127
	<30 SAR	Ref.	—	Ref.	—
Source of tobacco	Social/other	0.84 (0.36–2.00)	0.701	0.88 (0.33–2.34)	0.804
	Public/commercial	Ref.	—	Ref.	—
Anti-tobacco messages	No	0.87 (0.46–1.66)	0.671	0.83 (0.41–1.69)	0.609
	Yes	Ref.	—	Ref.	—
Perceived others’ smoking harmful	No	1.04 (0.51–2.14)	0.913	0.83 (0.38–1.79)	0.636
	Yes	Ref.	—	Ref.	—

Primary multivariable model: *n* = 174 (age, gender, grade, spending, message exposure, perceived harm). The adjusted OR for source of tobacco is from a sensitivity model additionally including it (*n* = 139); source is reported separately because it was recorded for only 227 participants. Bold: *p* < 0.05. Survey-weighted logistic regression with PSU-clustered (Taylor-linearised) variance. Outcome pools attempt to stop across all tobacco product types.

## 4 Discussion

This study obtained important insights into youth tobacco cessation behavior that both complement and diverge slightly from nationally reported estimates (6, 7). The study population was composed of 15-year-old boys current tobacco users enrolled in the third intermediate grade, representing the largest demographic subgroup.

Approximately one-quarter of the surveyed youth subgroup (23.1%) reported receiving some form of help or advice to support their efforts to quit, compared with (21.4%) reported in the WHO/CDC fact-sheet data, which are restricted to current smokers. In contrast, (79.3%) of participants reported making a quit attempt within the preceding 12 months, which was higher than the corresponding WHO/CDC estimate of (64.9%), also restricted to current smokers. The observed discrepancies may therefore reflect differences in analytic strata, survey weighting, and sample composition rather than true differences in cessation behavior. Such variation between weighted and unweighted estimates is well recognized in population-based survey research (19). The differences in the handling of design effects, including weighting, clustering, and stratification, can alter variance estimates and point estimates alike, in particular for outcomes with variable distributions such as the reported quitting attempt behavior, which is context-dependent (20).

Younger adolescents and first-intermediate students had higher odds of receiving cessation support, while those obtaining tobacco from social and or other sources were less likely to receive help. The unexpected association between perceiving others’ smoking as non-harmful and greater receipt of help may reflect differences related to peer influence, social identity, perceived attractiveness and desire to group belonging hindering adolescence from receiving help to quit (21–23). Socially sourced tobacco may therefore indicate greater integration into smoking-permissive networks, where tobacco use is normalized and cessation support may be less likely to be offered (21, 24). However, because our study did not directly measure peer norms, social support, or help-seeking behavior, this interpretation warrants further investigation. Within the sensitivity analysis, the association of receiving cessation support with tobacco source was attenuated and became non-significant, with no covariate remaining independently associated with receipt of help. These findings suggest that the observed associations may be sensitive to adjustment for tobacco source and the reduced sample size. This may reflect the reduced sample size (*n* = 139), which limited statistical power and increased imprecision of the estimates. Therefore, the absence of significant associations in this sensitivity model should be interpreted with caution.

In the multivariable analysis, 13-year-olds had significantly higher odds of having attempted to quit tobacco than 15-year-olds. Higher tobacco spending (≥30 SAR) showed a greater odd without significant association to quitting attempt, while tobacco source, gender, grade, anti-tobacco message exposure, and perceived harm were not associated with quit attempts. Although the association with higher tobacco spending (≥30 SAR) did not reach statistical significance. This positive trend may indicate that adolescents with greater tobacco expenditure are more likely to recognize the financial burden of tobacco use and consequently attempt cessation. However, higher spending may also reflect greater tobacco consumption or dependence. Further investigation using more direct measures to identify tobacco consumption, dependence and spending patterns.

The indicators, articulated within Levesque et al.’s Access-to-Health-Care Framework, for the ability to perceive domain, highlight important gaps in perceiving the harmful effect of others smoking also, more than half of them did not have teaching class about the dangers of tobacco. Even though 23.1% reported receiving some form of help quitting, still 20.7% did not report quitting attempt. This pattern raises concerns with respect to an excising passive service models that rely on individual willingness, as they may inadequately reach smokers with lower readiness, limited self-efficacy, and weaker social support, thereby more inequities in access. Unlike adult smokers, youth smokers are generally unaware of their smoking dependence. So, raising their awareness of their smoking dependence and providing them with strategies to resist peer pressure can increase self-efficacy and will encourage them to benefit from tobacco-cessation services (23).

With respect to tobacco accessibility and affordability, retail stores, shops, and public sources were the predominant sources of access, while most participants reported relatively low weekly expenditure (<30 SAR) on cigarettes and other tobacco products. Findings provide indirect indicators of the social and economic environment in which young people obtain and use tobacco and may therefore help contextualize potential barriers to cessation support. Informed by Levesque et al.’s Access-to-Health-Care Framework, access is not limited to the physical availability of services. Given that it is difficult to assess access to service delivery independently from users and the related social and environmental factors (25). In this context, the predominance of retail stores, shops, and other public sources as reported sources of tobacco suggests relatively accessible tobacco availability, while the predominance of weekly expenditure below 30 SAR provides an indication of the economic circumstances surrounding tobacco use. These measures should not be interpreted as direct measures of access to cessation services; rather, they serve as proxy indicators of the availability and affordability of tobacco within the wider environment. As such environmental accessibility may undermine cessation efforts by making tobacco readily obtainable and affordable, potentially reducing the practical barriers to continued use while cessation services may remain less visible or less readily accessed.

To overcome social and economic related factors, social marketing strategies are a well-recognized model used to apply control to social and environmental factors attributed to smoking and/or tobacco product access (23, 26). Among individuals aged 18–30 years, the Coronary Artery Risk Development in Young Adults (CARDIA) Longitudinal Study found that a US$1 increase in the price of a pack of cigarettes was associated with a 16% higher likelihood of initiating a quit attempt, followed by sustained smoking cessation (27). This supports the potential role of tobacco price increases as a population-level strategy to encourage quit attempts and promote sustained smoking cessation among adult and hence younger population.

As for the ability to engage, a considerable proportion of respondents lacked support for quitting or advice to quit; this requires deeper understanding, as it has the potential to negatively impact quitting attempts. Also, subgroup analysis showed that smoking students in the first year of intermediate schooling and aged 13 years had significantly higher odds of receiving help with quitting or advice to quit. Findings after adjustment for confounders concluded that not perceiving others’ smoking as harmful remained associated with higher odds of receiving help. This pattern suggests that risk awareness especially regarding the passive smoking among younger smokers may influence help-seeking. The findings indicate the need for targeted cessation strategies for older youth who are using tobacco and individuals with unclear perceptions of tobacco-related harm.

Older adolescents’ tobacco users who are uncertain about the harmful effect are critically underserved population who receive disproportionately less cessation support. Policies should mandate that school-based health programs begin no later than the early intermediate grades to systematically integrate structured cessation counseling and referral pathways, which can ensure that no student is overlooked. Furthermore, the significant association between uncertainty concerning tobacco harms and reduced quit attempts shows a clear knowledge gap that must be addressed. Awareness campaigns should be redesigned to deliver unambiguous, evidence-based messaging concerning the harms of tobacco use that is tailored specifically to adolescent audiences. Such interventions may act as a critical gateway to subsequent engagement with cessation services and may help increase quit attempts among tobacco users (26, 28, 29).

Our findings showed that smoking cessation support and quitting attempts among youth smokers are likely to be influenced by multiple interrelated dimensions of access outlines by Levesque et al.’s Access-to-Health-Care Framework. Receiving help or advice to quit was strongly associated with uncertainties related to smoking harmful effect. Quitting attempts are driven by younger intermediate school students. Although many youths may be motivated to stop smoking, their use of formal cessation services can be limited by social and service-related barriers. Qualitative evidence indicates that young people’s quitting behavior is influenced by family and peer relationships, personal attitudes, and concerns about the accessibility, acceptability, or stigma of cessation support (30). These findings underscore the importance of applying frameworks such as Levesque et al.’s Access-to-Health-Care Framework when evaluating tobacco-cessation support services, as they enable a comprehensive assessment of both service accessibility and youths’ ability to identify, seek, reach, afford, and engage with available services.

Youth smoking cessation is a complex behavior shaped by more than knowledge alone. A recent systematic review identified nicotine dependence, psychological factors, attitudes toward smoking, family and peer influences, and tobacco control policies as important predictors of quit attempts and abstinence among young people. These factors may partly explain why a number of youth attempt to quit but do not quit to achieve cessation (31). Also, it is worth considering that free access to cessation services alone is insufficient to ensure equity (32).

### 4.1 Limitations

Complex sample regression analysis appropriately accounts for the GYTS sampling design, including weighting, clustering, and stratification; however, it cannot eliminate all sources of bias. Complex-samples regression only adjusts for measured covariates; therefore, residual confounding arising from unmeasured factors, such as nicotine dependence, peer influence, parental smoking, school-level cessation resources, and motivation to quit, may still influence the observed associations. Thus, the findings should be interpreted as weighted population-level associations rather than definitive causal effects. Second, all measures were self-reported, so it is possible that responses were subject to recall or social desirability bias, particularly for sensitive behaviors related to tobacco acquisition and cessation attempts. Third, proxy indicators were adopted to operationalize access dimensions in the Levesque et al. framework; this approach, while conceptually justified, might not adequately reflect the complexity of structural and contextual factors that hinder cessation support. Finally, antismoking messages are provided to youth in both public health and school settings; thus, the findings in this study cannot be directly linked to health system performance alone.

## 5 Conclusion

The relatively low proportion of youth reporting receipt of cessation support (23.1%), despite the high proportion reporting quit attempts (79.3%), suggests a substantial gap between adolescents’ efforts to quit and their access to or engagement with effective cessation support. This highlights the need to strengthen proactive, youth-centered cessation outreach through schools, communities, and digital platforms, while ensuring equitable access across the dimensions of care.

Considering tobacco accessibility and affordability alongside receipt of cessation support have provided a broader understanding of the social, environmental, and individual factors shaping cessation behavior. However, future studies should use multiple validated measures to capture the individual domains of Levesque et al.’s Access-to-Health-Care Framework more comprehensively.

Ultimately, effective youth tobacco control requires an integrated approach that combines regulation of tobacco availability and affordability with stronger health literacy, early identification of adolescents who are reluctant to seek help, and proactive, tailored cessation interventions that engage young tobacco users to quit.

## Data Availability

Publicly available datasets were analyzed in this study. This data can be found at: The dataset (SPSS file), including all derived and newly coded variables used in the current analysis, is available from the corresponding author upon reasonable request. The GYTS is available through this link https://extranet.who.int/ncdsmicrodata/index.php/catalog/966.
