# Supplementary material for: Toward equity in smoking cessation: evidence from Saudi Arabia’s 2022 Global Youth Tobacco Survey through the Lens of Levesque’s Access-to-Health-Care Framework
Source: Front Public Health. 2026 Sep 23;14:1914890. doi: 10.3389/fpubh.2026.1914890 (PMC13642597; doi:10.3389/fpubh.2026.1914890)
Supplement: Supplementary file 1 [file Supplementary_file_1.DOCX]

Representative AI prompts used during manuscript development

1-“Help refine the research aim for a manuscript using the 2022 Saudi Global Youth Tobacco Survey to examine access to smoking cessation support among intermediate school students.”

2-“Suggest possible ways to frame inequities in youth access to smoking cessation services using Levesque et al.’s access-to-care framework, focusing on demand-side factors.”

3-“Improve the wording of this abstract for clarity, academic tone, and logical flow without changing the study findings or statistical results.”

4-“Rewrite the background and conclusion to emphasize the gap between free cessation service availability and actual access among adolescent smokers in Saudi Arabia.”

5-“Suggest concise phrasing for describing complex survey analysis that accounts for weighting, clustering, and stratification in the Saudi GYTS dataset.”

6-“Help interpret findings showing that younger students and those who had not attempted to quit had lower odds of receiving cessation help or advice.”

7-“Suggest public health implications based on findings that cessation support is underutilized among youth despite the availability of free services.”

8-“Edit the conclusion to highlight proactive, school-based cessation support, harm-awareness messaging, and targeted outreach for youth smokers.”

9-“Check whether the abstract maintains consistency between the purpose, methods, results, and conclusion sections.”

10-“Suggest a title and keywords for a manuscript examining determinants of inequitable access to smoking cessation services among Saudi youth using GYTS data.”

11-“Walk me through weighted and unweighted estimate using SPSS”
